# Stage-Specific Stability of Spray-Freeze-Dried Ovalbumin-Loaded Liposomes: Dissecting the Impact of Atomization, Freezing, and Drying on Cryoprotectant Performance

**DOI:** 10.3390/pharmaceutics18101338

**Published:** 2026-10-21

**Authors:** Xi Cheng, Mengyuan Li, Shen Yan, Shengyu Zhang, Xiao Dong Chen, Winston Duo Wu

**Affiliations:** Engineering Research Centre of Advanced Powder Technology, School of Chemical and Environmental Engineering, College of Chemistry, Chemical Engineering and Materials Science, Soochow University, Suzhou 215123, China; xicheng@alu.suda.edu.cn (X.C.); myli123@stu.suda.edu.cn (M.L.); syzhang0505@suda.edu.cn (S.Z.);

**Keywords:** spray-freeze-drying, unit operation, liposomal powder, ovalbumin, cryoprotectant

## Abstract

**Objectives**: Aqueous liposomal suspensions suffer from inherent physical and chemical instability, motivating the development of solid powder formulations. Spray-freeze-drying is a promising solidification technique; however, systematic investigations that isolate the impact of individual unit operations—atomization, freezing, and drying—on liposomal stability remain scarce. This study aimed to elucidate the stage-specific effects of each unit operation on the critical quality attributes of liposomal powders. **Methods**: Ovalbumin-loaded liposomes (O@L) prepared from DSPC, HSPC, or DPPC were processed using a self-developed microfluidic jet spray freezing tower coupled to a freeze dryer, with trehalose or mannitol as cryoprotectants. The effects of phospholipid composition, freezing temperature (−30 °C, −50 °C, and −70 °C), and freeze-drying cycle were assessed through the z-average, zeta potential, and encapsulation efficiency (EE) of the reconstituted liposomes, as well as the morphology, crystallinity, residual moisture, and hygroscopicity of the resulting powders. **Results**: Atomization induced protein leakage from the trehalose formulations, whereas mannitol better preserved EE. During freezing, trehalose provided superior cryoprotection, as mannitol partially crystallized and failed to uniformly coat the vesicles. After drying, however, vesicles frozen at −70 °C exhibited fusion attributable to non-uniform cryoprotectant distribution; a moderate freezing temperature (−50 °C) combined with a stepwise primary drying program proved optimal. Trehalose-based powders displayed higher residual moisture and irreversible crystallization above ~60% RH, whereas crystalline mannitol ensured low, reversible hygroscopicity. **Conclusions**: The optimal condition for a single unit operation does not guarantee the best overall outcome; rational spray-freeze-drying process design requires balancing stage-specific effects.

## 1. Introduction

Proteins and peptides have been extensively explored as therapeutics and vaccines owing to their exceptional potency and target specificity [1]. However, their clinical translation is frequently hampered by poor conformational stability, short plasma half-lives, and limited membrane permeability. Liposomal drug delivery systems have emerged as a promising solution to these challenges, offering sustained release and high biocompatibility [2,3]. Nevertheless, most commercial liposomal products are formulated as aqueous suspensions, which are inherently susceptible to physical stress (e.g., aggregation and leakage upon temperature fluctuations) and chemical degradation (e.g., phospholipid oxidation and hydrolysis) [4,5]. Consequently, such liquid formulations require buffering agents and preservatives, as well as an uninterrupted cold chain during storage and transport. Developing solid powder formulations has therefore become a pivotal strategy for overcoming the intrinsic instability of liposomes [6].

Spray-freeze-drying is an innovative technology that synergistically combines the advantages of spray–drying (direct particulate formation) and freeze-drying (preservation of thermolabile drugs without thermal degradation) [7,8]. Compared with spray–drying, in which hot-gas evaporation causes droplet shrinkage and thermal stress, spray-freeze-drying instantly freezes the droplets—locking in their geometry and leaving a porous matrix upon sublimation—and thus yields particles with higher porosity and superior bioactivity [9]; relative to freeze-drying, it offers precise particle size control while substantially shortening the process—atomized droplets freeze within milliseconds to seconds (vs. the 2–5 h bulk freezing of conventional freeze-drying), and their small size and high porosity shorten the vapor diffusion path, thereby reducing the overall drying cycle [10]. The process comprises three fundamental stages: atomization of the precursor liquids into droplets, rapid freezing of the droplets into frozen spheres, and sublimation drying [11]. Atomization greatly expands the interfacial surface area, facilitating rapid heat and mass transfer, and the resulting droplet size directly dictates the ultimate particle size [12,13]. During freezing, ice crystal growth and freeze-concentration impose severe stress on lipid bilayers, often triggering vesicle fusion and cargo leakage—processes governed largely by the freezing rate [4]. Previous studies have demonstrated the advantages of spray-freeze-drying for liposomal dry powders: for instance, Yin et al. [14] prepared redispersible rhEGF-loaded liposomal powders by ultrasonic spray-freeze-drying with well-preserved microstructure, owing to the small ice crystals generated during rapid freezing and the uniform distribution of cryoprotectants. It should be noted that, in such conventional processes, freezing is achieved by direct contact with liquid nitrogen (LN_2_, boiling point ≈ −196 °C); the extremely large temperature difference between LN_2_ and the atomized droplets renders freezing near-instantaneous and uncontrolled, lacking precise temperature control and making it difficult to systematically elucidate temperature-dependent freezing effects on liposomal integrity [15]. In the subsequent sublimation step, dehydration intensifies interactions among acyl chains, increasing bilayer density and driving vesicle aggregation [4,16]. To mitigate these process-induced stresses, cryoprotectants are routinely incorporated into the formulations.

Polyols and oligosaccharides, particularly trehalose and mannitol, are widely employed as cryoprotectants during spray-freeze-drying [17,18,19,20,21]. Trehalose, characterized by a high glass transition temperature (*T*_g_), high water solubility, and low toxicity, effectively stabilizes both liposomes and biological macromolecules. Its protective action is underpinned by two complementary mechanisms: the water replacement hypothesis and the vitrification hypothesis [4,17,22]. According to the former, trehalose forms hydrogen bonds with the phosphate headgroups of phospholipids, thereby replacing bound water, maintaining inter-headgroup spacing, lowering the phase transition temperature (*T*_m_), and preserving membrane integrity during dehydration [23,24]. Under the vitrification hypothesis, trehalose forms a highly viscous glassy matrix that physically immobilizes lipid bilayers, preventing vesicle fusion and shielding the vesicles from ice crystal damage [25,26,27]. By contrast, mannitol is a stable, non-hygroscopic, crystalline sugar alcohol that provides robust structural support as a bulking agent, although it offers limited direct protection against freeze–thaw stresses [28].

Moreover, conventional spray-freeze-drying relying on direct LN_2_ contact suffers from poor batch-to-batch reproducibility and uncontrolled particle attributes such as size, morphology, and porosity. To address these limitations, our group independently developed a novel microfluidic jet spray freezing tower (MFJSFT), in which droplets are frozen by temperature-controlled cold air (−30 °C to −70 °C in this study), thereby offering a moderate and tunable temperature difference that enables freezing temperature to be systematically varied [29]. Although extensive formulation screening has been reported for protein-loaded liposomes, systematic investigations that isolate the specific impact of each processing stage—atomization, controlled freezing, and drying—on liposomal stability remain scarce.

In this study, using ovalbumin (OVA)—a low-cost, well-characterized model hydrophilic protein—we systematically deconvoluted the spray-freeze-drying process into its three-unit operations—atomization, freezing, and drying—to elucidate the individual contribution of each stage to the critical quality attributes of liposomal powders. OVA-loaded liposomes (O@L) were processed with the MFJSFT coupled to a freeze dryer, and the effects of phospholipid phase transition temperature (DSPC, HSPC, and DPPC), freezing temperature (−30 °C, −50 °C, and −70 °C), and freeze-drying program were evaluated, with trehalose or mannitol added at 5% *w*/*v* relative to the aqueous phase. The resulting microparticles were characterized in terms of morphology, crystallinity, density, and moisture content, while the protective efficacy of the cryoprotectants was assessed by monitoring the average hydrodynamic diameter, zeta potential, and encapsulation efficiency of the reconstituted liposomes.

## 2. Materials and Methods

### 2.1. Materials

Ovalbumin (OVA, ~44.5 kDa) and the micro-BCA protein assay kit were purchased from Shanghai Yuanye Bio-Technology Co., Ltd. (Shanghai, China). Hydrogenated soy phosphatidylcholine (HSPC), 1,2-dioctadecanoyl-sn-glycero-3-phosphocholine (DSPC), and 1,2-dipalmitoyl-sn-glycero-3-phosphocholine (DPPC) were obtained from AVT (Shanghai) Pharmaceutical Technology Co., Ltd. (Shanghai, China). Cholesterol (CH), mannitol, and methanol were purchased from Sigma-Aldrich (Shanghai) Trading Co., Ltd. (Shanghai, China). Chloroform was obtained from Jiangsu Qiangsheng Functional Chemical Co., Ltd. (Suzhou, China). Trehalose dihydrate was obtained from TCI (Shanghai) Chemical Industry Development Co., Ltd. (Shanghai, China). Anhydrous formamide and one-component pyridine-free Karl Fischer reagent (5 mg H_2_O/mL) were purchased from Honeywell Co., Ltd. (Shanghai, China), and Shanghai Macklin Biochemical Technology Co., Ltd. (Shanghai, China), respectively. Ultrapure water (18.2–18.3 MΩ·cm) was generated using a Milli-Q Direct-16 water purification system (Merck Millipore, Darmstadt, Germany).

### 2.2. Preparation of the O@L Precursor Liquid

OVA-loaded liposome (O@L) precursor liquids were prepared via the thin-film hydration method followed by high-pressure homogenization. Three neutral saturated phospholipids with varying acyl chain lengths and main *T*_m_ were employed: DPPC (C16:0, *T*_m_ = 41 °C), HSPC (C16:0–C18:0, *T*_m_ = 50 °C), and DSPC (C18:0, *T*_m_ = 55 °C). Briefly, the selected phospholipid and CH were dissolved in a chloroform/methanol mixture (1:1, *v/v*) at a 2:1 molar ratio in a 100 mL round-bottom flask. A uniform lipid film was formed via rotary evaporation (N-1300D-WB, EYELA, Tokyo, Japan) at 60 rpm for 60 min under reduced pressure. The resulting film was hydrated with 100 mL of an aqueous OVA solution (1.0 mg/mL) at a temperature maintained 5 °C above the respective phospholipid’s *T*_m_ (i.e., 46 °C for DPPC, 55 °C for HSPC, and 60 °C for DSPC). The hydrated multilamellar vesicle (MLV) suspension was subjected to bath sonication (XJ-480HA, Fangxu Technology Co., Ltd., Shanghai, China) at 40 kHz for 20 min. After dissolving the designated cryoprotectant (trehalose or mannitol, concentration of 5% *w*/*v*), the suspension was passed through a high-pressure homogenizer (NANO2000, Antuos Nanotechnology (Suzhou) Co., Ltd., Suzhou, China) equipped with a microfluidization module at 1400 bar for 20 cycles to obtain the target O@L precursor liquid. The composition of the precursor liquids is shown in Table 1.

### 2.3. Fabrication of O@L Dry Powder

Liposomal microparticles were produced using a custom-designed MFJSFT integrated with a two-fluid nozzle. The tower chamber consists of four stacked stainless-steel cylinders (inner/outer diameter: 360/660 mm; height per cylinder: 400 mm), cooled by a low-temperature refrigerant that controls the wall and chamber temperatures. The process parameters are shown in Table 1. In each run, 100 mL of the precursor liquid was delivered via a syringe pump (SPLab01, Shenchen Precision Pump Co., Ltd., Baoding, China) at a constant flow rate of 5.0 mL/min and atomized into the cold-air freezing chamber. The chamber temperature was controlled at −30 ± 5 °C, −50 ± 5 °C, or −70 ± 5 °C. The frozen ice-balls were collected at the bottom tray and immediately transferred to a freeze dryer (DGJ-30H, Shanghai Bodeng Biological Science and Technology Co., Ltd., Shanghai, China) operating at a chamber pressure below 10 Pa. Primary drying was evaluated under three thermal protocols:

- Cycle 1 (Stepwise): −40 °C for 12 h, −20 °C for 12 h, and −10 °C for 12 h.
- Cycle 2 (Low-temperature isothermal): −40 °C for 36 h.
- Cycle 3 (Moderate-temperature isothermal): −20 °C for 36 h.

All protocols were followed by a secondary drying phase executed sequentially at 0 °C, 10 °C, and 20 °C for 8 h at each temperature step.

### 2.4. Dynamic Light Scattering (DLS)

The average hydrodynamic diameter (z-average) and zeta potential of the liposomes in the precursor liquids and in the reconstituted frozen and dried samples were measured using a Zetasizer Nano ZS90 (Malvern Instruments Ltd., Worcestershire, UK) at 25 °C. Prior to analysis, frozen samples were thawed at room temperature, and dried powder samples were reconstituted with ultrapure water; both were then diluted to a total lipid concentration of 1.0 mg/mL. The same reconstitution protocol was applied to all samples.

### 2.5. Differential Scanning Calorimetry (DSC)

Thermal transitions of the precursor liquids were evaluated using a differential scanning calorimeter (DSC 2500, TA Instruments, New Castle, DE, USA). Aliquots (~30 mg) were hermetically sealed in aluminum liquid pans, with an empty pan as the reference. Samples were equilibrated at 25 °C for 10 min, cooled to −70 °C at 10 °C/min, held isothermally for 10 min, and subsequently reheated to 25 °C at 2 °C/min under a continuous nitrogen purge (50 mL/min).

### 2.6. Protein Content and Encapsulation Efficiency (EE)

The EE of OVA within liposomes was determined indirectly by separating unencapsulated (free) protein. The O@L suspension was centrifuged at 13,000× *g* for 20 min at 4 °C using a high-speed refrigerated centrifuge (VORTEX 22 K, Hunan Xiangyi Instrument Development Co., Ltd., Changsha, China). The supernatant containing unencapsulated OVA was harvested, and its protein concentration was quantified using a micro-BCA assay kit via a microplate reader (M5, Molecular Devices, San Jose, CA, USA) at an absorbance wavelength of 562 nm (linear range: 10–200 μg/mL). The EE was calculated according to Equation (1):

(1) $$\mathrm{EE}=\frac{W_{\mathrm{total}}-W_{\mathrm{free}}}{W_{\mathrm{total}}}$$

where *W*_total_ represents the total mass of OVA loaded into the precursor formulation, and *W*_free_ denotes the mass of unencapsulated OVA detected in the supernatant.

### 2.7. Scanning Electron Microscopy (SEM)

Surface morphology and microstructural features of the spray-freeze-dried (SFD) powders were examined using a scanning electron microscope (SEM; SU1510, Hitachi Ltd., Tokyo, Japan) operated at an accelerating voltage of 5 kV. Powder samples were mounted onto aluminum stubs using double-sided conductive carbon tape and sputter-coated with gold (15 mA, 30 s) using an ion sputter coater (MC1000, Hitachi High Technologies Corp., Tokyo, Japan) prior to imaging.

### 2.8. Crystallographic Characterization

The crystalline structure of the samples was characterized using an X-ray diffractometer (XRD; D2 PHASER, Bruker, Germany) equipped with a Cu-Kα radiation source (λ = 1.54056 Å, operating at 30 kV and 10 mA). Diffraction patterns were collected over a 2θ range of 5° to 90° with a step size of 0.05° and a scan speed of 0.3 s per step.

### 2.9. Density and Moisture Content

The density of the samples was determined gravimetrically using a 5 mL graduated cylinder (precision: 0.1 mL). Initially, the empty cylinder was weighed, and the mass was recorded as *M*_0_. Subsequently, a predetermined mass of sample powder was carefully transferred into the cylinder, and the combined mass of the cylinder and sample was recorded as *M*_1_. The occupied volume of the powder was read as the bulk volume (*V*_1_). The cylinder was then sealed with parafilm to prevent powder loss during agitation. Manual tapping was performed for more than 200 cycles until the sample volume remained constant, and the resulting volume was recorded as the tapped volume (*V*_2_). All measurements were conducted in triplicate. The bulk density (*ρ*_b_), tapped density (*ρ*_t_), and Carr’s index (CI)—employed as an indicator of powder flowability—were calculated according to Equations (2), (3), and (4), respectively. The flowability was classified based on the CI values as follows: CI < 15%, excellent flowability; 15% ≤ CI ≤ 25%, intermediate flowability; and CI > 25%, poor flowability [30].

(2) $$\rho_{b}=\frac{M_{1}-M_{0}}{V_{1}}$$

(3) $$\rho_{t}=\frac{M_{1}-M_{0}}{V_{2}}$$

(4) $$\mathrm{CI}=\frac{\rho_{t}-\rho_{b}}{\rho_{t}}$$

The water content of the prepared samples was determined by volumetric Karl Fischer titration using a V10S titrator (Mettler Toledo, Greifensee, Switzerland). Briefly, 40 mL of methanol and 20 mL of formamide were introduced into the titration cell, and the system was automatically titrated with Karl Fischer reagent until the drift value decreased below 100 mV/min. Calibration was subsequently performed using a standard solution containing 5% *w*/*w* water (prepared by mixing 0.95 g of anhydrous methanol and 0.05 g of ultrapure water). Following completion of the calibration, the sample was introduced into the titration cell, the exact mass of the added sample (in grams) was entered into the instrument, and the titration was initiated. Upon completion, the measurement data were recorded. All determinations were carried out in triplicate.

### 2.10. Moisture Sorption Analysis

The hygroscopicity of the SFD powders was analyzed by dynamic vapor sorption (DVS) using a DVS-Advantage instrument (Surface Measurement Systems, London, UK). Approximately 5–20 mg of sample was placed in a sample pan and positioned on one side of the DVS microbalance, whereas an empty pan was loaded on the opposite side as a reference. The analysis was performed at a controlled temperature of 25.0 ± 0.1 °C with a nitrogen flow rate of 200 sccm. The relative humidity (RH) program was set as follows: the RH was initially increased from 0% to 30% RH in 5% RH steps, further increased from 30% to 90% RH in 10% RH steps, and finally decreased from 90% to 0% RH in 10% RH steps. At each step, equilibrium was considered to be reached when the dm/dt value changed by less than 0.005% within 5 min.

### 2.11. Statistical Analysis

Student’s *t*-test, one-way or two-way analysis of variance (ANOVA) was employed for statistical analysis. *** *p* < 0.001, ** *p* < 0.01, * *p* < 0.05 were considered significant.

## 3. Results and Discussion

### 3.1. Supercooling Behavior of the Precursor Liquids

**Figure 1:**
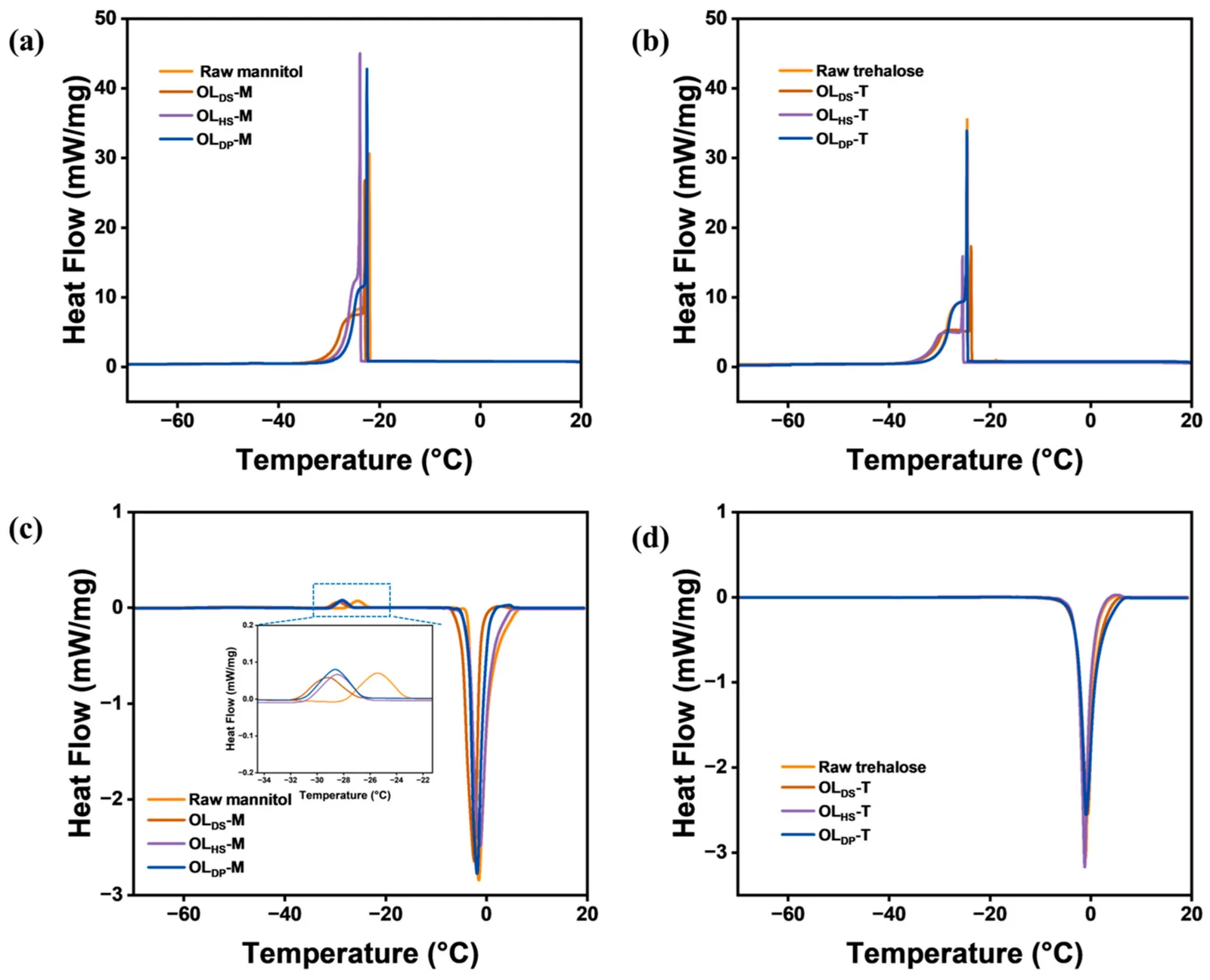
DSC cooling and reheating profiles of the mannitol- and trehalose-containing precursor liquids: (**a**) mannitol, cooling; (**b**) trehalose, cooling; (**c**) mannitol, reheating; (**d**) trehalose, reheating.

The cooling and subsequent rewarming behaviors of the precursor liquids were characterized by DSC. During cooling from 25 °C to −70 °C at 10 °C/min, the pure mannitol solution and the mannitol-containing precursor liquids (OL_DS_-M, OL_HS_-M, and OL_DP_-M) exhibited exothermic peaks at −21.9 °C, −22.6 °C, −23.5 °C, and −22.2 °C, respectively (Figure 1a), corresponding to the freezing of supercooled water [31]. During the subsequent heating stage (2 °C/min), these samples showed exothermic peaks at approximately −25.4 °C, −29.1 °C, −28.5 °C, and −28.6 °C, respectively, which were assigned to the cold crystallization of amorphous or metastable mannitol [32]. Upon further heating, an endothermic peak appeared at ~−2.7 °C, corresponding to ice melting (Figure 1c). Notably, the cold crystallization peak of mannitol shifted toward lower temperatures in the liposome-containing precursor liquids compared with the pure mannitol solution, indicating that the presence of liposomes modifies the crystallization behavior of mannitol [33].

For the trehalose-containing precursor liquids (OL_DS_-T, OL_HS_-T, and OL_DP_-T), analogous exothermic freezing peaks were observed at −23.7 °C, −25.1 °C, and −24.2 °C in the cooling cycles (Figure 1b), and an endothermic ice-melting peak appeared at ~−2.7 °C during the heating stage (Figure 1d). These peaks closely matched that of the pure trehalose solution, indicating that the presence of liposomes has little influence on the freezing behavior of trehalose, in contrast to the mannitol system. No distinct trehalose crystallization peak was detected, consistent with the well-documented tendency of trehalose to remain amorphous [32]. Across both cryoprotectant systems, the freezing temperatures of the liposome-containing precursor liquids were slightly lower than those of the corresponding pure sugar liquids, plausibly because the binding of water molecules at the liposomal bilayer interface increases the degree of supercooling and raises the nucleation barrier [34]. In addition, no discernible differences in DSC thermal behavior were observed among the DSPC, HSPC, and DPPC formulations, presumably owing to the relatively low mass fraction of liposomes in the system, such that the interfacial effects remained below the threshold required to produce distinguishable thermal signatures.

### 3.2. Impact of the Atomization Step on O@L Stability

**Figure 2:**
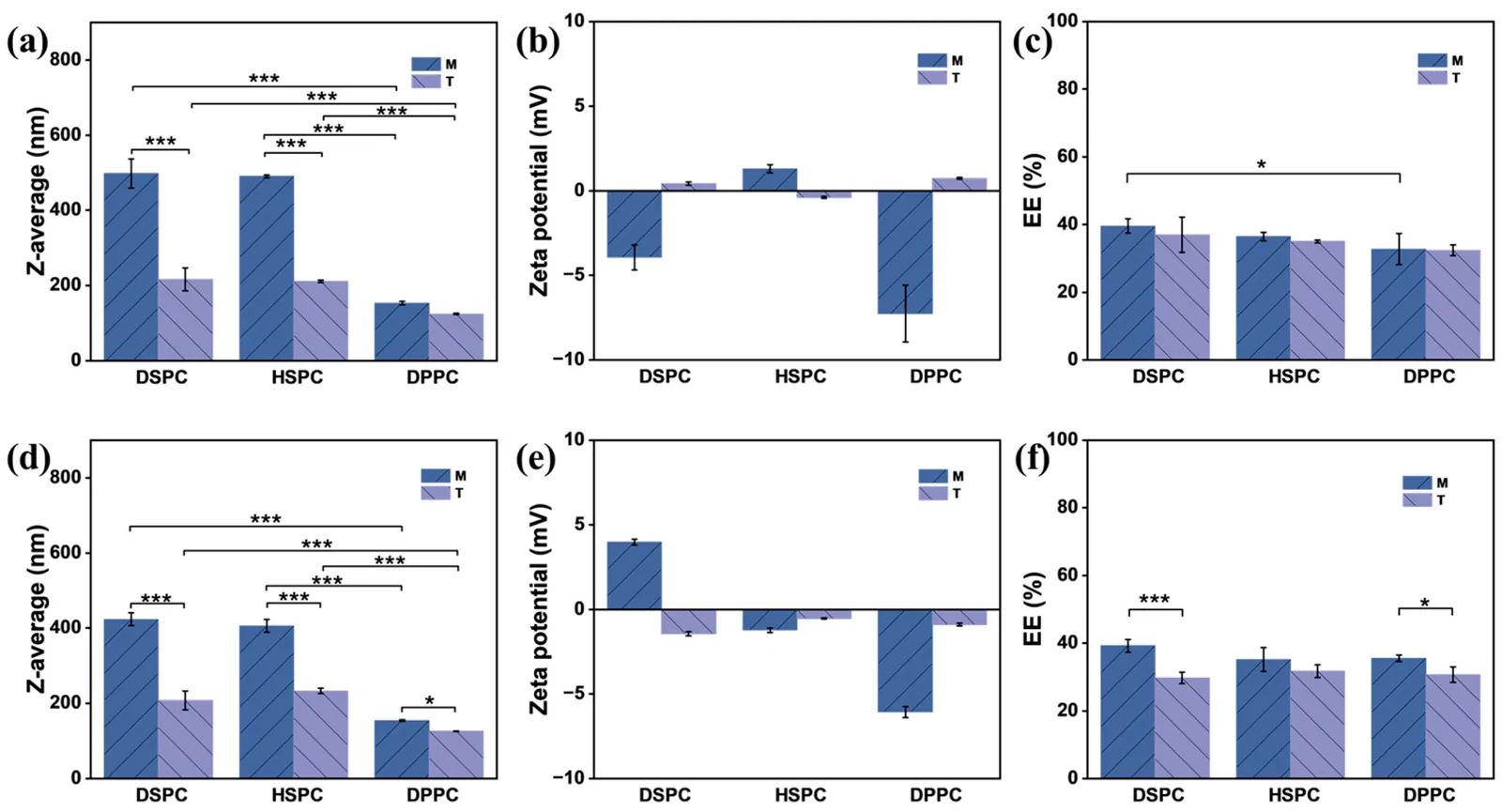
Effect of atomization on the z-average, zeta potential, and EE of O@L formulations with mannitol or trehalose as the cryoprotectant, before (**a**–**c**) and after (**d**–**f**) atomization (mean ± SD, *n* = 3, *** *p* < 0.001, * *p* < 0.05).

To isolate the effect of atomization from that of the subsequent freezing step, the atomized droplets were collected directly at the outlet of the two-fluid nozzle into a glass vessel before entering the cold-air chamber of the spray freezing tower, and were analyzed without having undergone freezing or drying. These samples represent the “after atomization” state and were compared with the corresponding precursor liquids (“before atomization”). Figure 2 presents the z-average, zeta potential, and EE of O@L before and after atomization. Before atomization, with respect to phospholipid type, the z-average of OL_DP_ was significantly smaller than those of OL_DS_ and OL_HS_ regardless of the cryoprotectant used (*p* < 0.0010), whereas no significant difference was observed between OL_DS_ and OL_HS_ (mannitol group, *p* = 0.8968; trehalose group, *p* = 0.9300). With respect to cryoprotectant type, for the DSPC and HSPC formulations, the z-average of the mannitol groups was significantly larger than those of the corresponding trehalose groups (both *p* < 0.0001), whereas no significant difference was observed for the DPPC formulation (*p* = 0.1093) (Figure 2a). These results indicate that trehalose favors the formation of O@L with smaller z-average, presumably because hydrogen bonding between trehalose and the phospholipid headgroups modulates the aggregation state of the vesicles. The DPPC formulation, which already exhibited a relatively small z-average, was less sensitive to the cryoprotectant type. Regarding EE before atomization, two-way ANOVA revealed no interaction between cryoprotectants and phospholipid type (*p* = 0.8242); the main effect of the cryoprotectant was not significant, whereas that of the phospholipid type was significant (*p* = 0.0246). Post hoc comparisons showed a significant difference between the DSPC and DPPC formulations only within the mannitol groups (*p* = 0.0470) (Figure 2c).

The phospholipid-dependent trends in z-average can be rationalized by the acyl chain structure of the lipids: DSPC is a pure C18:0 phospholipid, whereas HSPC is a mixed-chain hydrogenated soybean phosphatidylcholine dominated by long-chain (C16–C18) saturated species; both form rigid, low-curvature bilayers that tend to assemble into larger and more heterogeneous vesicles. DPPC, with its shorter C16:0 acyl chains, forms bilayers with a higher intrinsic curvature and therefore smaller equilibrium vesicle sizes. Because all DLS measurements were performed at 25 °C—below the *T*_m_ of all three phospholipids—the observed size differences reflect the intrinsic packing properties of the acyl chains rather than membrane fluidity at the measurement temperature. The zeta potentials of all precursor formulations were near neutral or slightly negative, consistent with the electrically neutral nature of DSPC, HSPC, and DPPC; the slight negative deviation may arise from the OVA (pI ≈ 4.7), which carries a net negative charge under the experimental pH conditions.

After atomization, two-way ANOVA revealed a significant cryoprotectant and phospholipid interaction (*p* < 0.0001). With respect to phospholipid type, the z-average of OL_DP_ remained significantly smaller than those of OL_DS_ and OL_HS_ irrespective of the cryoprotectant (*p* < 0.0001), with no significant difference between OL_DS_ and OL_HS_ (mannitol group, *p* = 0.3306; trehalose group, *p* = 0.1216). For the same phospholipid, the mannitol formulations exhibited significantly larger z-average values than their trehalose counterparts, although the difference between the two cryoprotectants was less pronounced for the OL_DP_ group (*p* = 0.0331) (Figure 2d). Overall, the ranking of z-average values after atomization mirrored that observed before atomization. Notably, atomization reduced the z-average of the mannitol formulations by approximately 80 nm, whereas that of the trehalose formulations remained essentially unchanged. Mannitol stabilizes liposomes mainly through physical separation [35], and the shear generated during atomization appears to disrupt loosely associated vesicle aggregates in the mannitol-containing precursor, leading to redispersion [23]; in contrast, the hydrogen-bonding network between trehalose and the phosphate headgroups confers strong structural cohesion that resists short-term airflow shear, thereby preserving a stable z-average distribution.

In contrast to the z-average, EE responded to atomization in a markedly different manner: after atomization, the EE of the trehalose formulations (29.7–31.8%) was significantly lower than that of the mannitol formulations (35.2–39.3%), whereas no difference in EE existed between the two systems before atomization, and the EE of the mannitol formulations was essentially unchanged by atomization (Figure 2f). These findings indicate that atomization induced leakage of the encapsulated OVA from the trehalose system, whereas the mannitol system exhibited superior encapsulation stability. A possible explanation is that, although the trehalose–phospholipid hydrogen-bonding network preserves vesicle integrity under shear, adsorption and interfacial shear at the gas–liquid interface during atomization compromise bilayer integrity, allowing the water-soluble OVA to escape; mannitol, by contrast, may limit vesicle exposure to the gas–liquid interface through physical separation, thereby retaining the encapsulated cargo. Taken together, the z-average and EE results indicate that the trehalose system favors a smaller z-average, whereas the mannitol system offers greater advantages in atomization stability and EE retention.

### 3.3. Impact of the Freezing Step on O@L Stability

**Figure 3:**
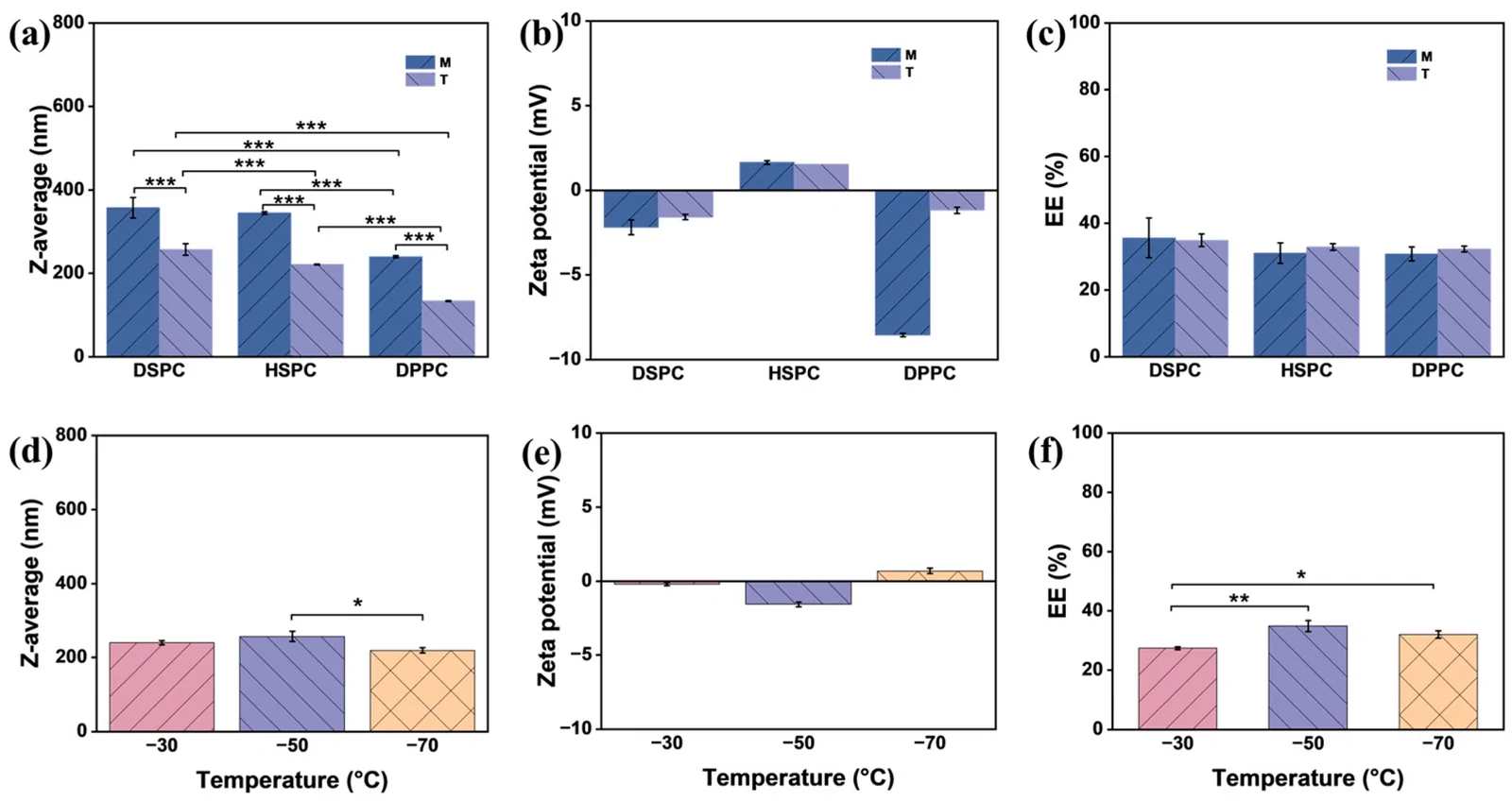
Effect of freezing temperature on the z-average, zeta potential, and EE of O@L formulations: (**a**–**c**) formulations with mannitol or trehalose as the cryoprotectant, frozen at −50 °C; (**d**–**f**) OL_DS_-T frozen at −30 °C, −50 °C, and −70 °C (mean ± SD, *n* = 3, *** *p* < 0.001, ** *p* < 0.01, * *p* < 0.05).

To systematically investigate the effect of the freezing step on the physical stability of O@L, the protective effects of mannitol and trehalose were first compared for the three O@L formulations at a freezing temperature of −50 °C; subsequently, using OL_DS_-T as a model system, the influence of lower and higher freezing temperatures (−30 °C and −70 °C) was further examined (Figure 3). Hereafter, spray-frozen samples are denoted as SF_−xx_, where xx indicates the freezing temperature (e.g., SF_−50_-OL_DS_-T).

At a freezing temperature of −50 °C, two-way ANOVA of the z-average revealed no interaction between cryoprotectant and phospholipid type (*p* = 0.2336). With respect to phospholipid type, the z-average of OL_DP_ remained significantly smaller than those of OL_DS_ and OL_HS_ regardless of the cryoprotectant (*p* < 0.0001), a ranking consistent with that observed before and after atomization (Section 3.2), indicating that freezing at −50 °C did not alter the inherent z-average hierarchy imposed by acyl chain length. With respect to cryoprotectant type, the mannitol formulations exhibited significantly larger z-average values than their trehalose counterparts under the same phospholipid composition (*p* < 0.0001) (Figure 3a); notably, unlike the results obtained before and after atomization, a significant cryoprotectant effect was also observed for OL_DP_ after freezing. This can be ascribed to the partial crystallization of mannitol during freezing (see the DSC results in Section 3.1): crystallization induces phase separation within the freeze-concentrated phase, preventing mannitol from uniformly coating the vesicles in an amorphous form and thereby weakening its ability to suppress membrane aggregation.

The superior cryoprotective performance of trehalose can be rationalized by the vitrification and water replacement hypotheses: trehalose forms a stable glassy matrix around the vesicles during freezing, markedly reducing their molecular mobility and suppressing membrane fusion and aggregation [36]; meanwhile, trehalose directly interacts with phospholipid headgroups via hydrogen bonding, stabilizing the lateral organization of the bilayer and reducing membrane permeability, thereby limiting drug leakage. Taking together, these results indicate that trehalose more effectively maintains liposomal stability during freezing.

The freezing temperature also exerted a significant influence on the stability of OL_DS_-T (Figure 3d–f). Post hoc analysis showed that the EE of SF_−30_-OL_DS_-T decreased to 27.47%, significantly lower than that of SF_−50_-OL_DS_-T (*p* = 0.0013) (Figure 3f). According to the DSC results (Section 3.1), the crystallization temperature of the OL_DS_-T precursor liquid was approximately −23.7 °C; thus, at a freezing temperature of −30 °C, the small degree of supercooling slowed ice crystal growth, ultimately yielding larger ice crystals. Meanwhile, the slower freezing rate also promoted the migration of trehalose toward the vesicle surface and its accumulation into a concentrated surface layer, which increased the osmotic pressure gradient across the bilayer, driving the outward efflux of the internal aqueous phase and causing partial drug leakage [15]. On the other hand, because ice crystal growth occurred mainly in the inter-vesicular spaces, ice formation within the liposomes was limited and the membrane sustained relatively little direct mechanical damage; consequently, no significant change in z-average was observed under this condition, and the reduction in EE was the dominant manifestation. When the freezing temperature was lowered to −70 °C, the z-average of SF_−70_-OL_DS_-T was significantly smaller than that of SF_−50_-OL_DS_-T (*p* = 0.0069) (Figure 3d), whereas no significant difference in EE was observed between the two temperatures. At this temperature, the high degree of supercooling and extremely fast freezing rate produced small, uniformly distributed ice crystals, markedly reducing mechanical damage to the vesicles; moreover, rapid freezing shortened the time available for trehalose migration and redistribution, preventing the osmotic imbalance caused by localized over-concentration. These results demonstrate that the freezing rate is a critical parameter governing the freeze stability of O@L, with faster freezing being more favorable for preserving its structural integrity. Nevertheless, the comparable EE values at −50 °C and −70 °C suggest that the benefit of increasing the freezing rate approaches a threshold at approximately −50 °C, beyond which further cooling confers only marginal improvements in OVA retention.

### 3.4. Impact of the Drying Step on O@L Stability

**Figure 4:**
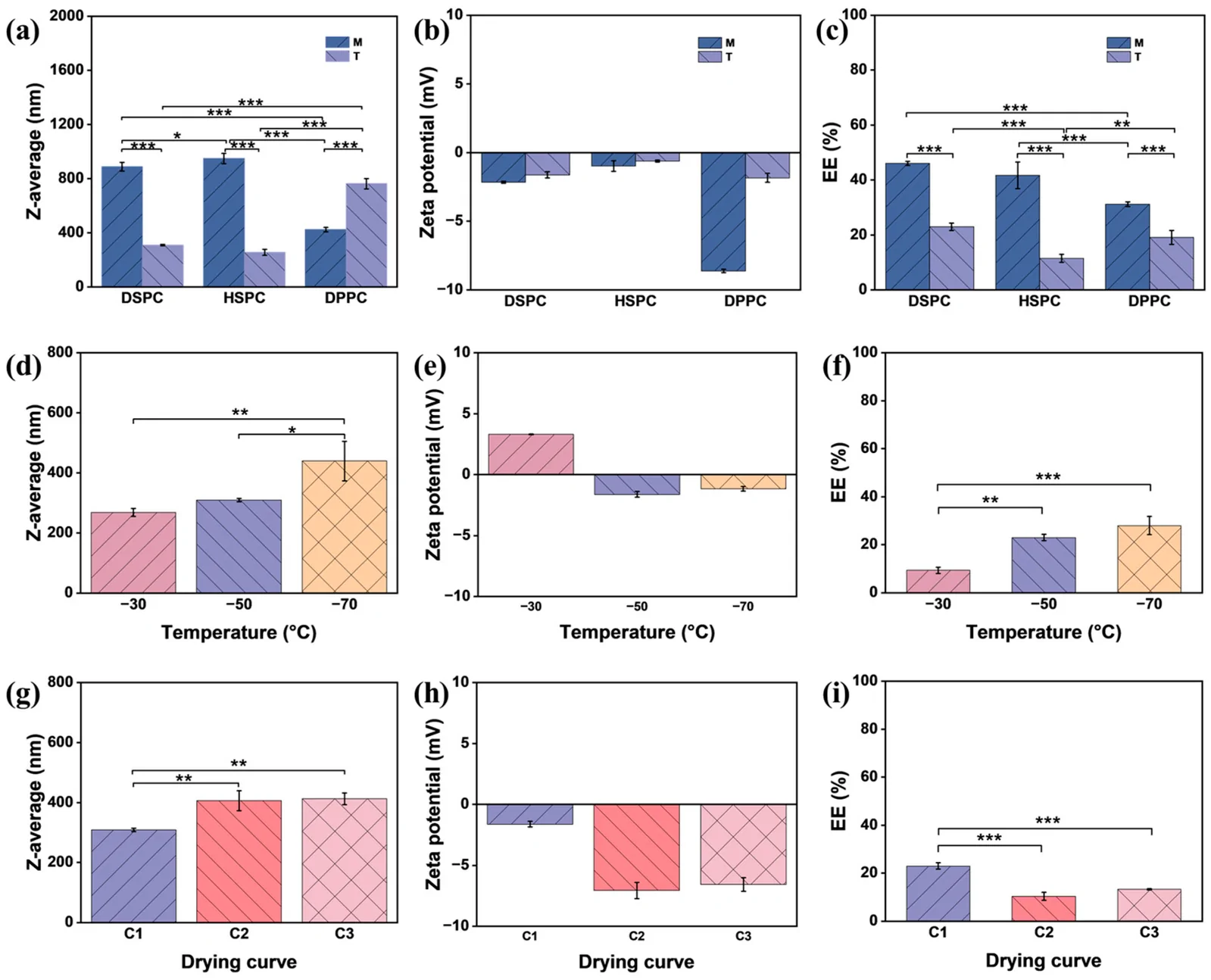
Effect of the freeze-drying cycle on the z-average, zeta potential, and EE of reconstituted O@L: (**a**–**c**) cryoprotectant comparison (mannitol vs. trehalose) at a freezing temperature of −50 °C (cycle C1); (**d**–**f**) OL_DS_-T spray-frozen at −30 °C, −50 °C, and −70 °C (cycle C1); (**g**–**i**) SF_−50_-OL_DS_-T dried with cycles C1–C3 (mean ± SD, *n* = 3, *** *p* < 0.001, ** *p* < 0.01, * *p* < 0.05).

Figure 4 presents the z-average, zeta potential, and EE of O@L after spray-freeze-drying under different conditions followed by reconstitution. Under freeze-drying cycle C1, two-way ANOVA revealed a significant interaction between cryoprotectant and phospholipid type (*p* < 0.0001). For the DSPC and HSPC systems, the mannitol formulations exhibited significantly larger z-average values than their trehalose counterparts (*p* < 0.0001), whereas the opposite was observed for DPPC (*p* < 0.0001); accordingly, OL_DP_ remained the smallest formulation with mannitol but became the largest with trehalose (both *p* < 0.0001). These results indicate that trehalose provides inadequate protection for DPPC liposomes during drying, whose small size and high membrane curvature renders them particularly vulnerable.

Regarding EE, the trend differed from that of the z-average: under the same phospholipid composition, the EE of the mannitol formulations was significantly higher than that of the trehalose formulations (*p* < 0.0001). Within the mannitol groups, the EE of OL_DP_ was significantly lower than those of OL_DS_ and OL_HS_ (*p* < 0.0001), with no significant difference between the latter two (*p* = 0.1032); within the trehalose groups, the EE of OL_HS_ was significantly lower than those of OL_DS_ (*p* = 0.0002) and OL_DP_ (*p* = 0.0057), whereas OL_DS_ and OL_DP_ did not differ significantly (*p* = 0.1530). Overall, OL_DS_ consistently achieved the highest EE irrespective of the cryoprotectant, while the second-best formulation depended on the cryoprotectant type—OL_HS_ with mannitol and OL_DP_ with trehalose.

Compared with the post-freezing state, the z-average values of all three mannitol formulations increased significantly after drying (OL_DS_: *p* = 0.0002; OL_HS_: *p* = 0.0060; OL_DP_: *p* = 0.0060) (Figure 3a and Figure 4a), indicating substantial vesicle aggregation and fusion. Notably, the apparent increase in EE observed under this condition should not be interpreted as improved protection; rather, the larger internal aqueous volume of the fused vesicles may allow a fraction of the previously leaked drug to be re-encapsulated, producing an apparent EE increase. Consistent with the DSC results (Figure 1c), mannitol partially crystallizes during freezing (cold crystallization at approximately −28 °C) and mainly provides mechanical support against macroscopic collapse during drying, while failing to protect the lipid bilayer against dehydration-induced damage or to suppress membrane fusion and aggregation. This observation is consistent with the conclusion of Susa et al. [16] that mannitol provides limited protection to liposomes during drying.

When trehalose was used as the cryoprotectant, the z-average of SF_−50_D_C1_-OL_DP_-T increased significantly relative to the post-freezing state (*p* = 0.0060) (Figure 3a and Figure 4a). Through its multiple hydroxyl groups, trehalose replaces water molecules and forms hydrogen bonds with the phosphate and ester groups of phospholipid headgroups, thereby maintaining the lateral spacing between headgroups, attenuating van der Waals interactions between hydrocarbon chains, and reducing the propensity for membrane fusion [4]. Nevertheless, the DPPC system is intrinsically small with high membrane curvature; under the capillary forces and interfacial stress generated by ice sublimation, such small vesicles are more susceptible to structural damage and aggregation, accounting for the relatively poor drying stability of OL_DP_ even under trehalose protection. After drying, the EE of the three trehalose formulations decreased to 23.00% (OL_DS_: *p* = 0.0074), 11.47% (OL_HS_: *p* = 0.0005), and 19.07% (OL_DP_: *p* = 0.0274) (Figure 3c and Figure 4c), indicating varying degrees of OVA leakage during drying and rehydration. The freeze-drying stability of liposomes is closely related to the properties of the encapsulated drug: OVA is a hydrophilic macromolecule localized mainly in the internal aqueous phase and is therefore prone to loss along with water removal. Consistently, the hydrophilic drugs methotrexate and doxorubicin have been reported to leak by 61.1% and 24.5%, respectively, during liposome freeze-drying—far more than the lipophilic drug tamoxifen [37]. Moreover, Garvey et al. [38], using X-ray diffraction and small-angle neutron scattering, demonstrated that liposomes may undergo phase transitions during rehydration that are accompanied by a transient increase in membrane permeability, which may further contribute to OVA leakage.

The freezing temperature also significantly affected the stability of the dried OL_DS_-T formulations. Post hoc analysis revealed that the EE of SF_−30_D_C1_-OL_DS_-T decreased to 9.34%, significantly lower than that of SF_−50_D_C1_-OL_DS_-T (*p* = 0.0012) (Figure 4f). As shown by Yang et al. [15], a slow freezing rate favors the formation of large ice crystals that directly puncture or compress the lipid bilayer, and such structural damage is further amplified during subsequent drying and rehydration [39]. Conversely, the z-average of SF_−70_D_C1_-OL_DS_-T increased to 439.3 nm, significantly exceeding that of SF_−50_D_C1_-OL_DS_-T (*p* = 0.0147), suggestive of membrane fusion (Figure 4d). Although rapid freezing produces fine ice crystals that minimize osmotic injury, an excessively fast freezing rate may compromise the uniformity of cryoprotectant distribution around the vesicles, leaving localized regions insufficiently protected [15]; according to the water replacement hypothesis, such heterogeneity weakens the ability of trehalose to maintain phospholipid headgroup spacing and to suppress membrane fusion [40]. Therefore, a moderate freezing rate (e.g., −50 °C) that balances ice crystal size against the uniformity of cryoprotectant distribution is expected to minimize the adverse effects of the drying step on O@L.

**Figure 5:**
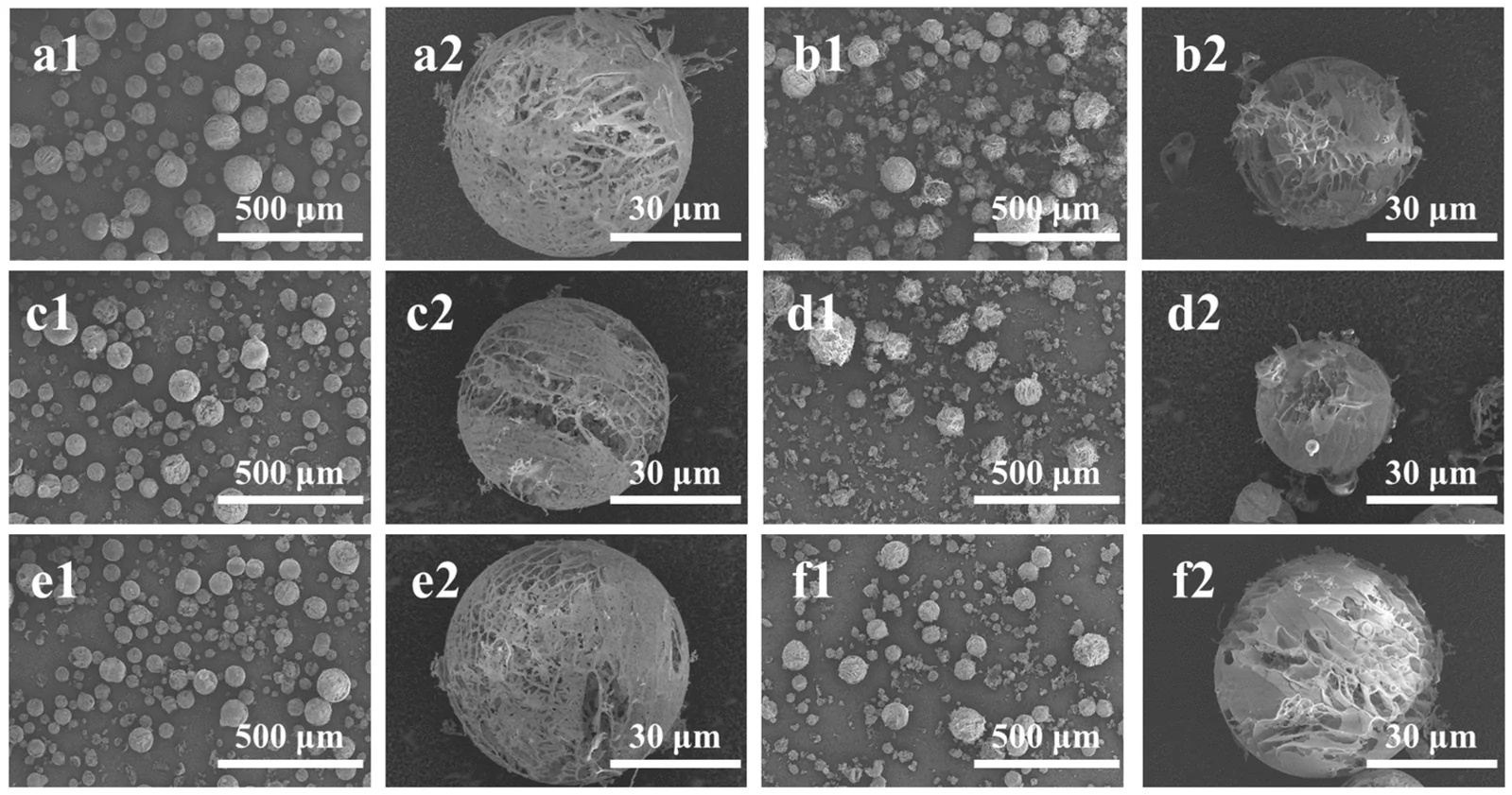
SEM images of SFD O@L microparticles formulated with different phospholipids ((**a1**,**a2**,**b1**,**b2**): DSPC; (**c1**,**c2**,**d1**,**d2**): HSPC; (**e1**,**e2**,**f1**,**f2**): DPPC) and cryoprotectants (mannitol: (**a1**,**a2**,**c1**,**c2**,**e1**,**e2**); trehalose: (**b1**,**b2**,**d1**,**d2**,**f1**,**f2**)), spray-frozen at −50 °C and dried with cycle C1. Subfigures 1 and 2 show the same sample, with the only difference being the scale bar (magnification).

Further adjustment of the primary drying parameters revealed a significant influence of the drying program on the stability of SF_−50_D-OL_DS_-T. Compared with cycle C1, the z-average values under cycle C2 (*p* = 0.0044) and cycle C3 (*p* = 0.0033) were significantly larger, and the EE values were significantly lower (C2: *p* < 0.0001; C3: *p* = 0.0002) (Figure 4g–i). Primary drying removes ice by sublimation, and the sublimation rate is governed by the heat input per unit time [41]. Cycle C2 employed a lower shelf temperature (−40 °C, 36 h), resulting in a slow sublimation rate and prolonged drying; the vesicles thus remained in a semi-frozen, highly viscous state for an extended period, which enhanced membrane permeation and aggravated drug leakage, while the prolonged metastable environment may also have promoted slow vesicle fusion and aggregation. Cycle C3 employed a higher shelf temperature (−20 °C, 36 h). Because ice sublimation consumes substantial latent heat, the actual product temperature remains considerably lower than the shelf setpoint only while ice is still present [41]; once sublimation is complete, the product temperature approaches the shelf temperature. According to the kinetic studies of Sun et al. and Franzè et al. [42,43] on liposome leakage and membrane fusion, when the product temperature approaches or slightly exceeds the *T*_g_′ of the freeze-concentrated matrix, the viscosity of the glassy phase decreases markedly and liposomes undergo fusion and leakage at low but non-negligible rates, even in the absence of particle collapse (Figure 5). Unlike cycle C1, in which the shelf was held at −20 °C and −10 °C only during the final 24 h—by which time most of the ice had already been removed at −40 °C and the freeze-concentrated matrix had largely vitrified—cycle C3 exposed the product to a comparatively high temperature throughout the entire 36 h primary drying, so that the cumulative time spent near or above *T*_g_′ was substantially longer. Accordingly, it is speculated that under cycle C3 the product temperature was closer to—or slightly above—*T*_g_′, weakening glassy-state protection and resulting in a stability loss similar to that observed under cycle C2. Looking ahead, the collapse temperature of the freeze-concentrated matrix will be determined by freeze-dry microscopy to verify this interpretation and to establish quantitative criteria for stage-specific freeze-drying design.

### 3.5. Physicochemical Properties of O@L Powder

**Figure 6:**
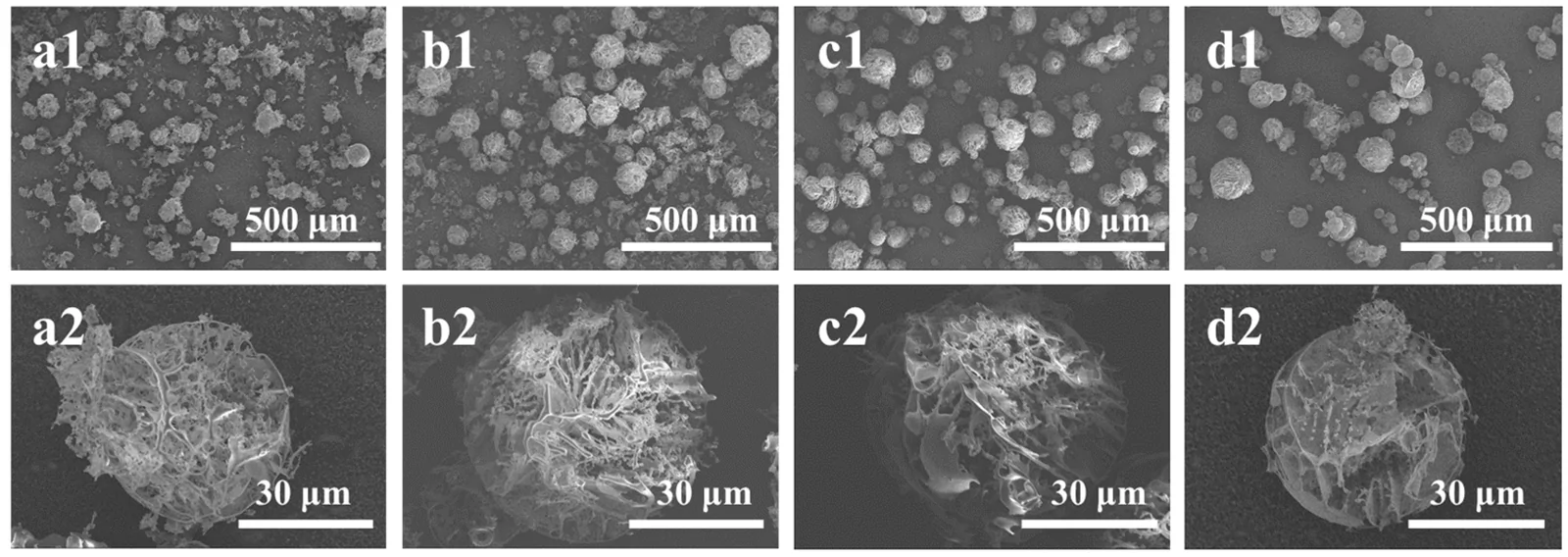
SEM images of SFD OL_DS_-T microparticles prepared under different process conditions: freezing temperatures of −30 °C (**a1**,**a2**) and −70 °C (**b1**,**b2**), and freeze-drying cycles C2 (**c1**,**c2**) and C3 (**d1**,**d2**). Subfigures 1 and 2 show the same sample, with the only difference being the scale bar (magnification).

The morphology of the microparticles obtained by spray-freeze-drying with different phospholipids and cryoprotectants was examined by SEM (Figure 5 and Figure 6). Overall, the microparticle morphology was governed mainly by the cryoprotectant type and freezing temperature, whereas the phospholipid composition had no discernible effect on the overall particle morphology. During spray freezing at −50 °C, ice crystals grew rapidly, and the cryoprotectant migrated with the advancing ice front and became enriched in the interstitial spaces between ice crystals; upon sublimation, mannitol left a distinct crystalline solute layer on the microparticle surface, yielding a loose and porous structure, whereas trehalose formed a partially continuous film with relatively dense pore walls (Figure 5).

At a freezing temperature of −30 °C, the low degree of supercooling prolonged ice crystal growth, producing large ice crystals and, consequently, dried microparticles with larger lamellar voids, thinner pore walls, reduced mechanical strength, and greater susceptibility to fragmentation (Figure 6(a1,a2)). When the freezing temperature was lowered to −50 °C and −70 °C (Figure 6(b1,b2)), the higher nucleation density and faster growth rate generated numerous fine ice crystals; after drying, the lamellar voids shrank and the microstructure became denser. Notably, no discernible differences in surface structure or overall morphology were observed between the −50 °C and −70 °C samples, indicating that, within this temperature range, the influence of freezing rate on microparticle morphology reaches a plateau. In addition, the microparticles obtained with freeze-drying cycles 2 (Figure 6(c1,c2)) and 3 (Figure 6(d1,d2)) showed no discernible morphological differences from those of cycle 1 (Figure 5(b1,b2)), indicating that, within the scope of this study, the primary drying conditions had a limited effect on microparticle morphology.

**Figure 7:**
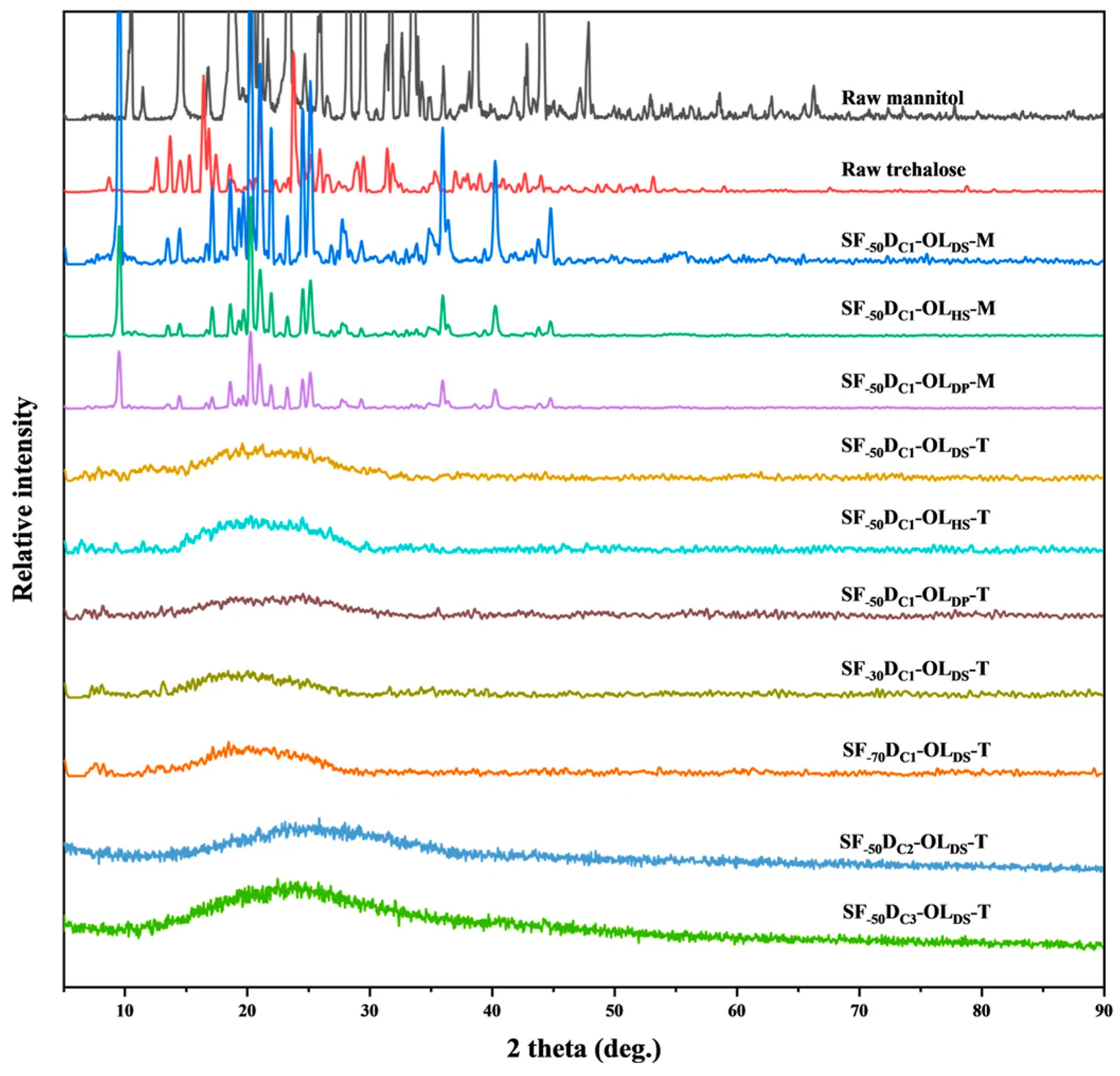
XRD patterns of SFD O@L samples.

The crystallographic properties of mannitol and trehalose, both as raw materials and as the corresponding SFD powders, were characterized by XRD (Figure 7). The diffractograms were governed predominantly by the type of cryoprotectant. Mannitol commonly exists in three polymorphic forms: the α-form exhibits characteristic diffraction peaks at 2θ = 13.8° and 17.2°, the β-form at 10.6°, 14.7°, and 16.8°, and the δ-form at 9.7° [44]. Under the rapid, non-equilibrium cooling conditions of spray-freeze-drying, crystallization is kinetically controlled, favoring the formation of the metastable δ-form, which has a lower nucleation barrier and a faster nucleation rate. The pronounced enhancement of the characteristic peak at 2θ = 9.7° in the XRD pattern provides direct evidence of this transition. As a crystallizing cryoprotectant, mannitol can provide structural support that suppresses macroscopic cake collapse; however, the phase separation and localized mechanical stress associated with crystallization may increase the risk of liposome aggregation [34], which is consistent with the increased z-average and the apparently elevated EE observed upon reconstitution. In contrast, no sharp diffraction peaks were detected for the trehalose-containing samples; only broadened, diffuse halos were present, indicating that the powders were in an amorphous state. As a non-reducing disaccharide, the water-substitution and vitrification-based protective mechanisms of trehalose rely precisely on the formation of this stable amorphous matrix during the freeze-drying process.

The physical properties of the SFD powders are summarized in Table 2. Two-way ANOVA revealed a significant phospholipid × cryoprotectant interaction for the bulk density (*p* < 0.001): within the mannitol formulations, the bulk density increased significantly in the order DSPC < HSPC < DPPC, whereas the trehalose formulations did not differ significantly. The CI of all samples exceeded 25%, indicating poor flowability, which may be associated with the porous, fragile structure of the microparticles, which are prone to fragmentation and adhesion under vibration [45]. The residual moisture content of the trehalose-containing samples (3.72–4.88%) was significantly higher than that of the mannitol-containing samples (*p* < 0.0001), which is attributed to the strong water-binding capacity of trehalose as a polyhydroxy amorphous excipient. The freezing temperature also influenced the residual moisture content: the −30 °C samples (4.29%) contained slightly less moisture than the −50 °C samples (4.79%), possibly because the larger ice crystals left wider pores after sublimation, lowering the mass transfer resistance and facilitating water removal [46]. In addition, the residual moisture content of the samples obtained with cycle C2 was 3.72%, significantly lower than that of C1 (*p* = 0.0039), possibly because at −40 °C the matrix remained in a rigid glassy state, which preserved the integrity of the freeze-concentrated phase, kept the sublimation channels open, and allowed secondary drying to proceed more thoroughly.

**Figure 8:**
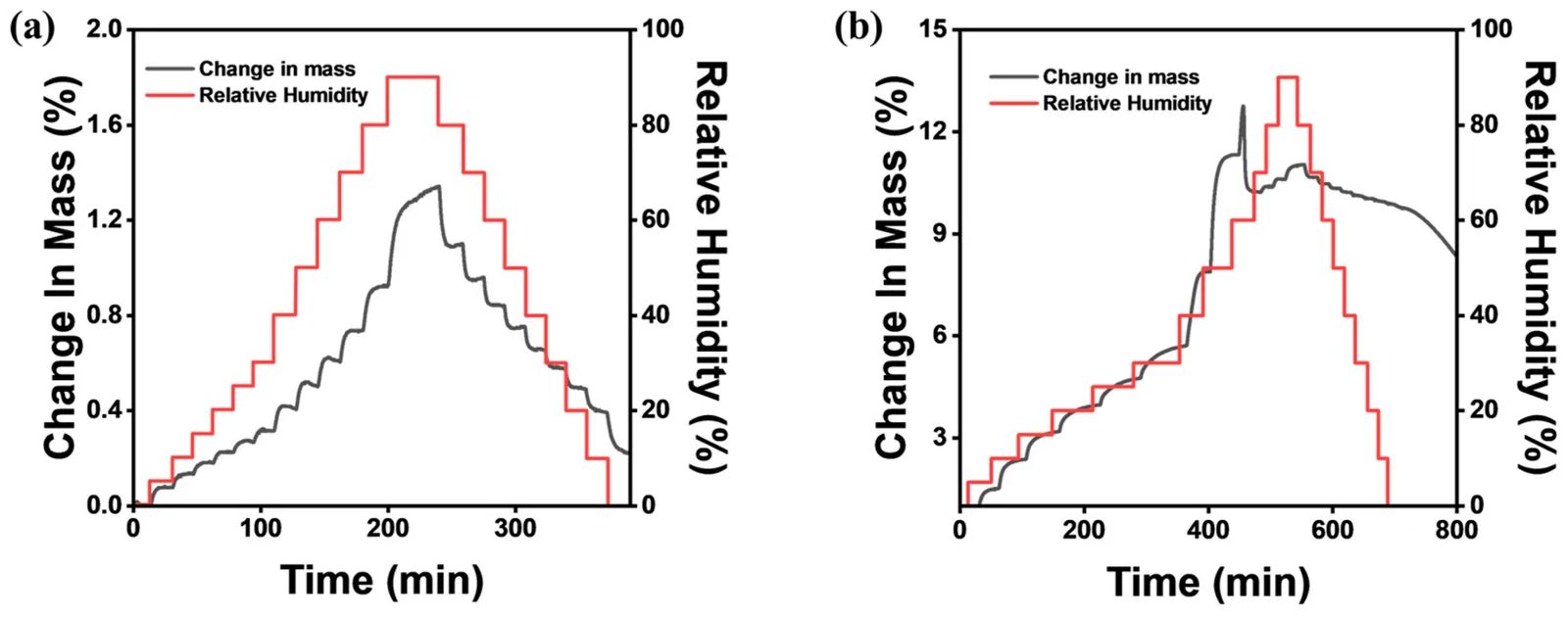
Moisture sorption and desorption behavior of SF_−50_D_C1_-OL_DS_-M (**a**), SF_−50_D_C1_-OL_DS_-T (**b**) at 25 °C.

The hygroscopic behavior of SF_−50_D_C1_-OL_DS_-M and SF_−50_D_C1_-OL_DS_-T at 25 °C was investigated by DVS over a range of relative humidities (Figure 8). SF_−50_D_C1_-OL_DS_-M gradually gained mass from 0% to 90% RH, and the mass progressively recovered as the humidity was stepwise reduced, essentially returning to its initial value at 0% RH, demonstrating reversible sorption behavior and extremely low hygroscopicity. This result is consistent with the crystalline nature of mannitol confirmed by XRD. In the crystal lattice, the hydroxyl groups of mannitol are largely engaged in intermolecular hydrogen bonding, which limits their accessibility to water molecules. Moisture uptake is therefore restricted mainly to surface adsorption, accounting for the low hygroscopicity observed. In contrast, SF_−50_D_C1_-OL_DS_-T exhibited typical amorphous-to-crystalline transition behavior. The sample absorbed moisture slowly between 0% and 40% RH, and the uptake accelerated between 40% and 50% RH. Rapid water absorption occurred between 50% and 60% RH, followed by a mass decrease at approximately 60% RH. This mass loss corresponds to the crystallization of amorphous trehalose, as the adsorbed water was expelled during crystal formation. After crystallization, the adsorbed free water was incorporated as water of crystallization, rendering the sorption process irreversible: as the RH decreased, the sample mass failed to return to its initial value and stabilized at a higher level [47]. This behavior originates from the conversion of amorphous trehalose into trehalose dihydrate crystals and represents a critical environmental sensitivity that warrants particular attention during the storage of trehalose-based SFD formulations [31,34].

## 4. Conclusions

In this study, the spray-freeze-drying process was systematically deconvoluted into its three-unit operations—atomization, freezing, and drying—to elucidate the individual contribution of each stage to the stability of O@L processed with a self-developed MFJSFT. The atomization step revealed a clear cryoprotectant-dependent trade-off: trehalose favored smaller vesicle z-average through hydrogen bonding with phospholipid headgroups, whereas mannitol better preserved EE, as gas–liquid interfacial shear during atomization induced OVA leakage from the trehalose system. During freezing, trehalose provided superior cryoprotection via vitrification and water replacement, whereas the partial crystallization of mannitol (cold crystallization at approximately −28 °C) compromised its ability to uniformly coat the vesicles. Within the freezing step itself, faster freezing better preserves liposomal integrity. During drying, however, stage-coupling effects emerged: the mannitol formulations underwent pronounced aggregation and fusion, and their apparent EE increase was attributed to re-encapsulation of leaked drug within fused vesicles rather than improved protection; meanwhile, vesicles frozen at −70 °C exhibited signs of fusion after drying, plausibly due to non-uniform cryoprotectant distribution at excessively fast freezing rates. A moderate freezing temperature (−50 °C) therefore proved optimal for the overall process, balancing ice crystal size against cryoprotectant distribution uniformity. Among the freeze-drying cycles, a stepwise primary drying program (C1) afforded the best stability: overly slow sublimation (C2, −40 °C) prolonged the semi-frozen state and aggravated leakage, whereas an excessive shelf temperature (C3, −20 °C) likely brought the product close to or above the *T*g′ of the freeze-concentrated matrix, weakening glassy-state protection. The phospholipid composition governed the inherent vesicle z-average (DPPC < DSPC ≈ HSPC) but had little influence on powder morphology, which was dominated by the cryoprotectant type and freezing temperature. The resulting powders exhibited poor flowability (CI > 25%); trehalose-based powders showed higher residual moisture and underwent irreversible crystallization to trehalose dihydrate above ~60% RH, whereas crystalline mannitol conferred reversible, low hygroscopicity. Overall, this stage-by-stage analysis demonstrates that the optimal condition for an individual unit operation does not necessarily translate to the optimal outcome for the entire SFD process. A freezing temperature of −50 °C combined with a stepwise primary drying program represents the most favorable processing window for O@L powders, and the cryoprotectant selection should be guided by the targeted balance between z-average, EE, and storage hygroscopicity. These findings provide a mechanistic framework for the rational process design of liposomal powders by spray-freeze-drying. It should be noted that these conclusions were drawn from OVA (~44.5 kDa) as a model hydrophilic protein cargo; their generalizability to cargos of substantially different molecular sizes, to other therapeutic proteins, or to lipophilic and nucleic acid-loaded systems require further verification. Building on this mechanistic framework, future work will explore binary trehalose–mannitol cryoprotectant systems that combine the cryoprotective advantages of both sugars, together with surfactant-containing formulations and systematically varied membrane compositions (e.g., phospholipid/cholesterol ratio, charged or PEGylated lipids). The storage stability and immunogenicity of the OVA-loaded powders will also be evaluated, and the stage-coupling effects identified herein will be addressed through design-of-experiments approaches that explicitly model interactions between unit operations, with benchmarking against conventional spray–drying and freeze-drying, toward quality-by-design-based process optimization.

## Figures and Tables

**Table 1:** Summary of the formulation compositions (phospholipid type and cryoprotectant) and process parameters (freezing temperature and freeze-drying cycle) of the O@L microparticles.

Sample	Composition of the Precursor Liquids								Process Parameters	
	DSPC (g)	HSPC (g)	DPPC (g)	Cholesterol (g)	OVA (g)	Mannitol (g)	Trehalose (g)	Water (mL)	Freezing Temperature (°C)	Drying Cycle
SF_−50_D_C1_-OL_DS_-M	0.160	-	-	0.039	0.100	5.000	-	100.00	−50	C1
SF_−50_D_C1_-OL_HS_-M	-	0.160	-	0.041		5.000	-		−50	C1
SF_−50_D_C1_-OL_DP_-M	-	-	0.158	0.042		5.000	-		−50	C1
SF_−50_D_C1_-OL_DS_-T	0.160	-	-	0.039		-	5.000		−50	C1
SF_−50_D_C1_-OL_HS_-T	-	0.160	-	0.041		-	5.000		−50	C1
SF_−50_D_C1_-OL_DP_-T	-	-	0.158	0.042		-	5.000		−50	C1
SF_−30_D_C1_-OL_DS_-T	0.160	-	-	0.039		-	5.000		−30	C1
SF_−70_D_C1_-OL_DS_-T	0.160	-	-	0.039		-	5.000		−70	C1
SF_−50_D_C2_-OL_DS_-T	0.160	-	-	0.039		-	5.000		−50	C2
SF_−50_D_C3_-OL_DS_-T	0.160	-	-	0.039		-	5.000		−50	C3

**Table 2:** Bulk density, tapped density, Carr’s index, and moisture content of the SFD O@L powders (mean ± SD, n = 3, samples 1–6 (freeze-dried at −50 °C with cycle C1) were compared by two-way ANOVA (phospholipid type × cryoprotectant type) followed by Tukey’s test; samples 4, 7, and 8 (freezing temperature series) and samples 4, 9, and 10 (drying cycle series) were each compared by one-way ANOVA followed by Tukey’s test. Values within each comparison series and column followed by different lowercase letters are significantly different (*p* < 0.05)).

No.	Sample	Bulk Density (g/cm^3^)	Tapped Density (g/cm^3^)	Carr’s Index (%)	Moisture Content (% w/w)
1	SF_−50_D_C1_-OL_DS_-M	0.012 ± 0.001 ^c^	0.027 ± 0.002 ^b^	56.09 ± 1.72 ^a^	2.36 ± 0.18 ^b^
2	SF_−50_D_C1_-OL_HS_-M	0.015 ± 0.001 ^b^	0.035 ± 0.003 ^a^	57.20 ± 1.68 ^a^	2.62 ± 0.17 ^b^
3	SF_−50_D_C1_-OL_DP_-M	0.017 ± 0.001 ^b^	0.038 ± 0.003 ^a^	54.95 ± 3.58 ^a^	2.54 ± 0.31 ^b^
4	SF_−50_D_C1_-OL_DS_-T	0.020 ± 0.001 ^a^	0.040 ± 0.007 ^a^	50.72 ± 2.05 ^a^	4.79 ± 0.33 ^a^
5	SF_−50_D_C1_-OL_HS_-T	0.018 ± 0.001 ^a^	0.040 ± 0.004 ^a^	54.06 ± 3.41 ^a^	4.88 ± 0.27 ^a^
6	SF_−50_D_C1_-OL_DP_-T	0.019 ± 0.001 ^a^	0.038 ± 0.002 ^a^	50.00 ± 1.77 ^b^	4.54 ± 0.16 ^a^
7	SF_−30_D_C1_-OL_DS_-T	0.019 ± 0.001 ^a^	0.041 ± 0.001 ^a^	53.44 ± 3.55 ^a^	4.29 ± 0.01 ^a^
8	SF_−70_D_C1_-OL_DS_-T	0.020 ± 0.001 ^a^	0.042 ± 0.002 ^a^	53.03 ± 2.14 ^a^	4.49 ± 0.25 ^a^
9	SF_−50_D_C2_-OL_DS_-T	0.019 ± 0.001 ^a^	0.042 ± 0.002 ^a^	53.69 ± 2.73 ^a^	3.72 ± 0.23 ^b^
10	SF_−50_D_C3_-OL_DS_-T	0.019 ± 0.001 ^a^	0.042 ± 0.002 ^a^	53.82 ± 1.02 ^a^	4.66 ± 0.11 ^a^

## Data Availability

Data presented in this study is contained within the article. Further inquiries can be directed to the corresponding authors.

## Abbreviations

The following abbreviations are used in this manuscript:

ANOVA	Analysis of variance
CH	Cholesterol
CI	Carr’s Index
DLS	Dynamic light scattering
DPPC	1,2-dipalmitoyl-sn-glycero-3-phosphocholine
DSC	Differential scanning calorimetry
DSPC	1,2-dioctadecanoyl-sn-glycero-3-phosphocholine
DVS	Dynamic vapor sorption
EE	Encapsulation efficiency
HSPC	Hydrogenated soy phosphatidylcholine
LN_2_	Liquid nitrogen
MFJSFT	Microfluidic jet spray freezing tower
MLV	Multilamellar vesicle
O@L	OVA-loaded liposomes
OVA	Ovalbumin
RH	Relative humidity
SEM	Scanning electron microscopy
SFD	Spray-freeze-dried
*T*_g_	Glass transition temperature
*T*_g_′	Glass transition temperature of the maximally freeze-concentrated matrix
*T*_m_	Phase transition temperature
XRD	X-ray diffractometer
